# Modeling collisions in laying hens as a tool to identify causative factors for keel bone fractures and means to reduce their occurrence and severity

**DOI:** 10.1371/journal.pone.0200025

**Published:** 2018-07-10

**Authors:** Michael Toscano, Francesca Booth, Gemma Richards, Steven Brown, Darrin Karcher, John Tarlton

**Affiliations:** 1 Center for Proper Housing: Poultry and Rabbits (ZTHZ), Division of Animal Welfare, VPH Institute, University of Bern, Zollikofen, Switzerland; 2 School of Veterinary Sciences, University of Bristol, Lower Langford, North Somerset, England; 3 Department of Animal Sciences, Purdue University, West Lafayette, Indiana, United States of America; Gaziosmanpasa University, TURKEY

## Abstract

Keel fractures represent a major productivity and welfare issue for the laying hen industry with greater than 50% of birds in recent surveys across various commercial operations and nations exhibiting some form of damage by end of lay. While the causes are likely multifactorial and influenced by age, diet, genetic line, and other factors, high energy collisions with house furnishings and conspecifics in the barn are believed to be a major contribution to the frequency and severity of factures. The current study applies a previously described *ex vivo* impact testing protocol to quantify susceptibility to keel bone damage across an extensive range of collision energies and ages. We also link fracture susceptibility with bone and physiological measures likely to influence skeletal resilience. Further, we applied the impact testing protocol to evaluate the benefit of an omega-3 enriched diet to improve bone health and reduce fracture susceptibility. Our results indicated that fracture susceptibility increased rapidly from 23 weeks of age, peaking at 49.5 weeks of age and thereafter decreasing. Fracture susceptibility also varied with multiple natural characteristics of bone, including mineral density, though the nature of that relationship was dependent on whether an old fracture was present. Severity of the experimental fracture demonstrated considerable variation with collision energy and biomechanical properties. An omega-3 enhanced diet provided a protective effect against fractures, though only in terms of collision energies that were relatively low. In conclusion, the impact testing protocol provided a unique means to assess fracture susceptibility and quantify the role of likely influencing bird-level biological factors, both those that vary naturally as well as when altered through specific interventions.

## Introduction

Keel fractures represent a major productivity and welfare issue for the laying hen industry with greater than 50% of birds in recent surveys across various commercial operations and nations exhibiting some form of damage by end of lay [1–7]. Keel bone damage is recognized as one of the most important problems by the United Kingdom’s Farm Animal Welfare Council [8,9], the EU Food Safety Authority [10], and a consortium of animal welfare researchers [11]. Recent findings showing that the presence of housing elements (e.g., perches, support beams, water lines) at greater elevations (and in greater number) correlated to the prevalence of fractures suggested that collisions during locomotion are a principal cause of fractures [1]. In this scenario, it is hypothesized that descent from greater heights results in an increased kinetic energy at impact and a consequent increased risk of fracture.

Previous work by our group sought to define the upper and lower boundaries of the energetic threshold for keel bone fracture and its relationship to key influencing factors, such as age and bone mineral density. The protocol used euthanized birds in an *ex vivo* model where collisions of quantifiable energetic loads here created with a drop-weight impact tester [12]. Results from this study indicated the protocol was effective as collision energy was associated with an increased likelihood of fracture in line with predictions. We have subsequently used the technique to demonstrate clear differences in fracture susceptibility between five distinct purebred/crossbred laying hen lines for two collision energies [13]. The current work sought to expand on these initial studies and examine fracture susceptibility across a broader range of collision energies and ages. We also sought to link susceptibility to keel fracture with likely influential bone and physiological measures. The first experiment (Impact Modeling) hypothesized that the likelihood of fracture (and degree of severity) increased with: age of the birds, collision energy, and decreases in bone mineral density, bone strength, flexibility, and calcium:phosphorus ratio as an indicator of new bone formation. In a second experiment (Omega-3 Assessment), we sought to demonstrate the potential for the impact testing protocol to assess induced changes in skeletal resilience in response to dietary omega-3. Our hypothesis was that birds provided diets high in dietary omega-3 would have reduced susceptibility to fracture across a range of collision energies compared to hens fed a control diet based on previous findings [7,14]. Furthermore, hens receiving the omega-3 enriched diets would have increased bone strength, flexibility and mineral content. All raw data is available as a Supplementary Information File, S1 Database.

## Materials and methods

### Experiment 1: Impact modeling

#### Animals

All procedures were approved by the University of Bristol’s Animal Use Committee (University Identification Number: UB/12/027). For the project, Hyline Brown laying hens were collected from five commercial hen houses (four of which were located on one site) in the UK owned and managed by an industrial partner (Noble Foods) within a five-month period. With the exception of one house used for hens 65 weeks of age that were barn-housed (i.e. same interior to the others but without range access), all flocks were provided range access from approximately 1000 to dusk. All houses contained similar internal furnishing, e.g., single tier with a raised slatted area and aerial perches, and subject to a company-wide management protocol defining nutrition, lighting regime etc. Five ages were selected (23, 34, 45, 55, and 65 weeks of age) to provide a comprehensive representation of the change in likelihood of fracture with age. We attempted to collect 80 birds at each age for the study, though this was not possible due to flock availability and logistical problems particularly at 23 and 45 weeks of age where only 40 hens were collected. Hens at 65 weeks of age were collected from a single house at a particular farm. The remaining ages were selected from either one or two of four possible houses at a single site (i.e., 23 weeks of age: 40 hens from House 1; 34 weeks of age: 40 hens each from House 1 and House 2; 45 weeks of age: 40 hens from House 4; 55 weeks of age: 40 hens each from House 4 and House 5). All birds were collected from stratified positions within the hen house and included an approximate equal number of samples with and without fracture as assessed by palpation [15]. Sampling from a single flock at certain ages is an experimental confound but was required to ensure hens were of the same age. Collected birds were placed in lairage crates and transferred to a holding facility near Bristol University’s Langford Veterinary Campus, a trip of between two and five hours depending on the farm location. Birds were maintained for a maximum of 60 hours within pens (2.5 * 3.0 m; approximately 3.5 hens/m^2^) at the holding facility with *ad libitum* access to feed, water, and straw (for use as bedding) until impact testing. The holding pens did not contain perches. Birds selected for impact testing on a given day were brought to the Langford Veterinary Campus in lairage crates, a journey of five minutes.

#### Impact Testing

Birds were euthanized using pentobarbital sodium (0.3 ml IV/bird; Euthosal, Pfizer, Switzerland), a method of killing which eliminates convulsions during the death process and the possibility of breaking bones inadvertently. Within thirty minutes of death, birds were weighed, palpated to assess existing keel damage (either old or new fractures), and placed supinely (keel facing upwards) at the base of a drop-weight impact testing apparatus (described in [10]). The impact load was dropped directly onto the keel approximately 2 cm cranial from the tip, the site where most fractures occur. Resulting collision energies were calculated on the basis that all potential energy (PE) was converted to kinetic energy (KE; J) (i.e., PE = KE = mass (kg) * 9.8 m/s^2^ *drop height (m)). Using a combination of seven pre-set drop heights and three metal plates of known mass mounted to the drop load, the protocol allowed for a possible 21 defined collision energies of which 16 were used during the experiment ranging from 0.934 to 14.304 J (Table 1).

**Table 1:** Number of animals impact tested for each combination of impact energy and age.

	Age (Weeks)					
Impact Energy (J)	23	30	42	50	60	Total (by Impact Energy)
0.934	4	4	3	4	3	18
1.022	0	2	0	0	0	2
1.081	0	4	0	5	5	14
2.43	5	6	6	8	7	32
2.657	0	2	0	0	0	2
2.812	0	6	0	8	8	22
3.833	4	8	6	8	10	36
4.19	0	1	0	0	0	1
4.435	0	7	0	8	11	26
5.983	6	8	7	7	10	38
6.924	0	5	0	7	8	20
8.601	9	7	6	5	7	34
9.953	0	5	0	7	4	16
11.499	8	6	6	5	4	29
13.307	0	5	0	5	2	12
14.304	4	4	3	3	0	14
Total (by Age)	40	80	37	80	79	

#### Bone and bird assessments

Following impact, birds were removed from the impact tester and scored for breast muscling using a three-point system where ‘0’ indicated poor and ‘2’ indicated substantial muscling. The system was designed so that the majority of hens would score as a ‘1’ as our primary interest was identifying birds of either extreme. The skin surrounding the keel was removed and breast muscle cut away from each side and weighed separately as a measure of consistency. The keel was then excised, examined for damage resulting from the impact testing procedure (hereafter referred to as an experimental fracture vs. existing old breaks), and given a score based on criteria previously reported [12]. Briefly, experimental fractures were classified as:

- **Minor**: evidence of a visible fracture line that was not continuous through the entire transverse (dorsal-ventral) axis of the keel. The keel remained intact as one continuous piece of bone.
- **Major**: visible fracture line observed as in minor fractures, but extending along the entire transverse axis with possible fracturing in other dimensions. The keel remained intact as one continuous piece of bone.
- **Severe fracture**: keels that had become completely separated into two distinct pieces of bone (i.e., as if by a shearing force), though mesentery or muscle could still connect the two sections.

Keels were also scored for old breaks and given a severity score as previously described [1,7]. The humerus was also removed at this point and stored at -20°C for later biomechanical assessment.

Following inspection for damage, the keels were prepared for further assessment using a bandsaw to cut each keel into three sections roughly equal in length along the coronal plane [12]. The middle section was then further separated into base and lateral sections in the coronal plane (along the intermuscular line [16]) and all samples frozen at -20°C until subsequent testing. The middle sections (i.e., base and lateral surface) were assessed for bone mineral density using dual energy X-ray absorptiometry (DEXA, Lunar PIXImus densitometer, Lunar Corp). To assess biomechanical properties of the humerus, each bone was loaded onto an Instron 6022 testing frame (Instron) and a constant force applied using a 3-point bending protocol [7]. The load and energy to reach structural failure (strength (N) and toughness (J), respectively, as defined by Melvin [17], as well as Young`s Modulus (mm/N; a measure of bone stiffness) was then calculated by the accompanying software. Assessment of biomechanical properties were distinct from the impact testing protocol where a rapid force is delivered to replicate a collision event. Humeri were pulverized and used to quantify Ca:P by dissolving freeze dried bone hydrolysate in distilled water (20 uL hydrosylate: 200 uL distilled water) and then analyzed (Konelab Prime 60i, Thermo Scientific).

### Experiment 2: Omega-3 assessment

All procedures were approved by the Cantonal Veterinary Office (Cantonal Approval: BE-57/14) and complied with Swiss regulations regarding the treatment of experimental animals. To provide an indication of impact testing’s utility in experimental settings, an omega-3 enriched diet provided by a second industrial partner (Alltech Nutrition, Inc) was assessed using the *ex vivo* protocol described above, though with several minor modifications. A custom designed and newly manufactured impact tester was used (Atelier Lorraine, Bern, Switzerland) and all experimental work was performed at the Aviforum Research Facility in Zollikofen, Switzerland. Two hundred and forty (N = 240) Lohmann Selected Leghorn (LSL) birds were utilized in total and received either one of two omega-3 enriched diets (1.5%, n = 80 birds; 3.0%, n = 80 birds) or a standard control diet (0.0%: n = 80 birds) (Tables 2 and 3). The omega-3 enriched diets were algae-based and developed by Alltech to provide relatively high concentrations of docosahexaenoic acid. Each diet was analyzed for omega-3 content by an independent laboratory that was blind to the relative omega-3 concentrations of the diet and confirmed the expected composition (Table 4). All birds were reared on-site and received a standard pre-layer diet until 18 weeks of age within a commercial barn (16.4 hens/m^2^ grid area only) containing a multi-tier aviary (Harmony 3, Landmeco A/S. Denmark). At 18 weeks of age, hens were transferred to a separate barn where they were group housed in a single pen (9.0*4.6 m; 5.8 hens/m^2^) containing 48 nipple drinkers, 18 meters of raised perches, and 6 round feeders (40 cm in diameter). At 23 week of age, birds were then divided equally among 12 pens (1.5*4.6 m; 20 birds/pen) containing eight nipple drinkers, three meters of raised perches, and one round feeder (40 cm in diameter) at floor-level. From 18 to 21 weeks of age, the hens were gradually transitioned from the pullet phase feed to the Control (0% diet) until all hens were receiving the Control diet. Beginning at 26 weeks of age and continuing a 7-day period, each pen was gradually transitioned to the pen-specific treatment diet (i.e., 1.5% or 3.0%) or maintained on the Control diet (0.0%). Diets were randomly assigned to pens. Throughout the study, researchers were blinded to which diet each pen was receiving.

**Table 2:** Nutrient and energy composition for the three treatment diets.

Nutrient	0.0%	1.5%	3.0%
Crude protein %	16.80	16.80	16.80
Poult ME kcal/kg	2800	2800	2800
Calcium %	3.90	3.90	3.90
Phos %	0.64	0.64	0.63
Avail Phos %	0.42	0.42	0.42
Fat %	4.06	4.78	5.49
Fibre %	2.30	2.26	2.23
Met %	0.42	0.42	0.42
Cys %	0.29	0.29	0.29
Me+Cys %	0.71	0.71	0.71
Lys %	0.86	0.87	0.87
Thr %	0.64	0.65	0.65
Na %	0.17	0.17	0.17
Cl %	0.27	0.27	0.27
K %	0.71	0.72	0.72
Linoleic acid %	1.74	1.73	1.71
Na+K-Cl (meq/kg)	181	181	182
DUA (meq/kg)	1892	1886	1881
Magnesium %	0.15	0.16	0.17
Sulphur %	0.16	0.16	0.16

**Table 3:** Mill formulations for treatment diets.

	Treatment Diet					
	0.0%		1.5%		3.0%	
Ingredient	%	Kg	%	Kg	%	Kg
Corn (8.4%)	62.89	943.4	61.55	923.3	60.21	903.2
Soybean meal (49%)	23.3	349.5	23.15	347.3	23	345.0
SP1 Algae	0	0.0	1.5	22.5	3	45.0
Soy oil	1.33	20.0	1.35	20.3	1.37	20.6
Monocalcium phosphate (21% P)	1.33	20.0	1.305	19.6	1.28	19.2
Limestone (coarse)	9.43	141.5	9.43	141.5	9.43	141.5
DL methionine	0.15	2.3	0.145	2.2	0.14	2.1
Salt	0.37	5.6	0.37	5.6	0.37	5.6
Vitamin/trace mineral premix	0.6	9.0	0.6	9.0	0.6	9.0
Vitamin E premix	0.4	6.0	0.4	6.0	0.4	6.0
Formic lactic acid	0.2	3.0	0.2	3.0	0.2	3.0
Total	100	1500.0	100	1500.0	100	1500.0

**Table 4:** Dietary composition (mg/g of milled feed) of selected omega-3 and omega-6s^†^.

	C18:2n6	C18:3n3	C20:4n6	C20:4n3	C20:5n3	C22:5n3	C22:6n3
Diet	LA	ALA	AA	ETA	EPA	DPA	DHA
0.0%	3186	264	0.55	0.00	3.05	2.51	7.61
1.5%	3168	250	0.80	3.99	3.65	5.30	280.67
3.0%	2891	251	1.39	7.24	6.23	7.96	498.44

^†^LA: Linoleic acid; ALA: α-Linolenic acid; AA: Arachidonic acid; ETA: Eicosatetraenoic acid; EPA: Eicosapentaenoic acid; DPA: Docosapentaenoic acid; DHA; Docosahexaenoic Acid.

#### Impact testing

Birds underwent impact testing at 36 and 45 weeks of age (i.e., after 9 and 18 weeks on the study diet, respectively) (3 diets*2 ages*40 birds/age/diet; N = 240 birds; 120 birds/time point). Immediately before impact, birds were euthanized with delivery of barbiturate (3 ml/bird, IP) and subsequent cervical dislocation. Within 120 seconds of death, bird mass was measured, birds loaded into the impact tester and a specific collision energy (ranging from 1.30 to 7.08 J) was delivered to a designated position 2 cm cranial from the tip. The difference in durations between testing and death for the two studies resulted from the different study locations, i.e., birds were able to be killed in the same room as the impact assessment for the Omega-3 study. Keel bones were removed following collisions, inspected for damage and assigned a categorical grade of fracture severity. The scoring system was the same as described above, though “Major” and “Severe” fractures were combined into a single classification (“Major”) to increase experimental power.

#### Bone properties, egg production and quality

Collected keels underwent a similar battery of testing as in Experiment 1, e.g., quantification of keel bone mineral density, though biomechanical testing was also performed at the base of the manubrial spine. Daily records of pen-level egg production were used to calculate the hen day percentage. In combination with weekly records for pen-level feed consumption, the ratio of feed consumption to egg production, as well as estimated amounts of calcium and specific omega-3 intakes were calculated. Egg mass and breaking strength as well as shell thickness and mass were evaluated by selecting three random eggs from each pen at four time points during the study (7, 9, 13, and 17 weeks after introduction of the treatment diets). The breaking strength (N) of the eggs was measured with an egg biomechanical testing frame (BMG 1.2mc/D, Fabr.Nr. MC 601/047, Messgerätebau Gutsch, Nauendorf, Germany). A piece of shell was selected at the widest circumference point and the thickness measured with a digital caliper. The entire shell was removed after emptying the egg contents and removing the interior membrane with a tweezers.

### Statistical analysis

#### Impact model

All analysis was done within MlwiN, a statistical software package specializing in multilevel analysis [18]. Results of impact testing were analyzed using logistic regression to evaluate the relationship between the occurrence of experimental fracture and predictors (i.e., week of age, collision energy, presence of old breaks, bird mass, mass of breast muscling, keel bone mineral density of the base and lateral sections, and humerus biomechanical properties (strength, toughness, stiffness)). All predictors with the exception of old break presence were considered as continuous variables. Due to multicollinearity concerns, the predictor week of age was analyzed separately from all others, though collision energy was included in both sets of analysis. Furthermore, separate models were run for all impact outcomes treating experimental fracture as a binary response and then again excluding those events where experimental fractures did not occur and fracture severity as a multinomial, categorical response. In other words, a total of four models were developed for each response. All predictors were initially included in the model and then removed individually when comparison of the respective Z-ratio with a standard normal distribution was greater than 1.96 (*P* > 0.05).

When appropriate, turning points (i.e., the point on the prediction line where the gradient of the function changes sign) were calculated by integrating the function and solving the equation for zero. We have included a histogram of sample sizes overlaying select figs to provide an indication of the number of responses across the range of predictors.

#### Omega-3 Assessment

Results of impact testing were analyzed as above using logistic regression to evaluate the relationship between the occurrence and severity of keel bone fracture and treatment diet, age at impact testing, and their interactions. Pen was the experimental unit with testing on the individual bird in a nested design where pen served as a blocking factor, i.e., 2 pens/diet/time point. All other responses (bone mineral density and biomechanical properties, bird mass, feed consumption, total eggs laid as the ratio of feed consumed to egg production, hen daily percentage by period and over the entire study) were analyzed using linear regression to determine the relationship between each response and diet, age, and their interaction. Diet and age were treated as categorical variables and omega-3 enriched diets (1.5%, 3.0%) compared to the 0.0% control diet. Means and standard deviations for all responses are provided across diet and age combinations. Additional data on intake of omega-3 and omega-6 content is also provided although was not statistically analyzed.

## Results

### Impact modeling

#### Binary response

**Fig 1:**
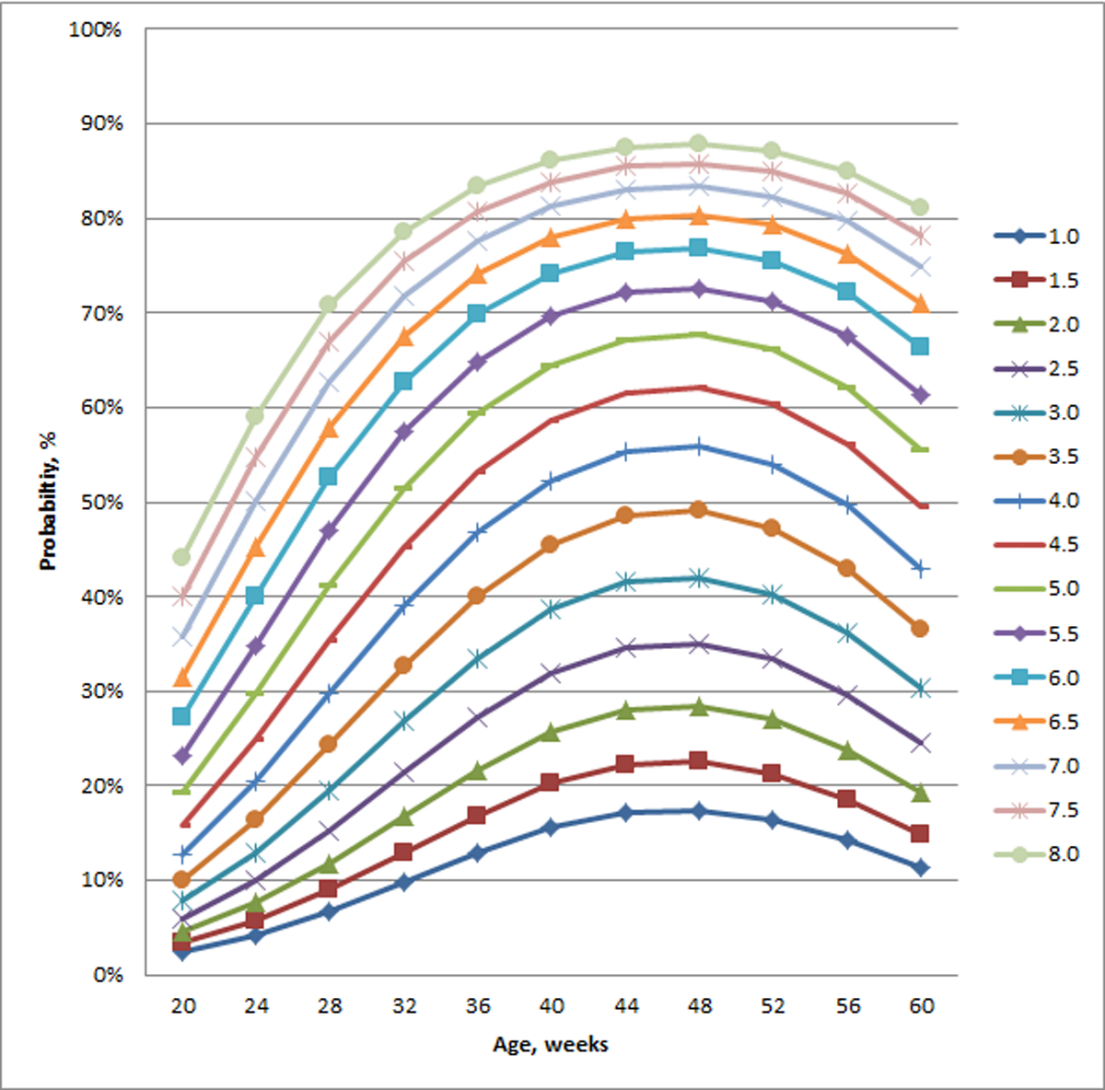
Modeled probability (% Y-axis) of experimental fracture in relation to hen age (weeks; X-axis) and collision energy (J).

**Fig 2:**
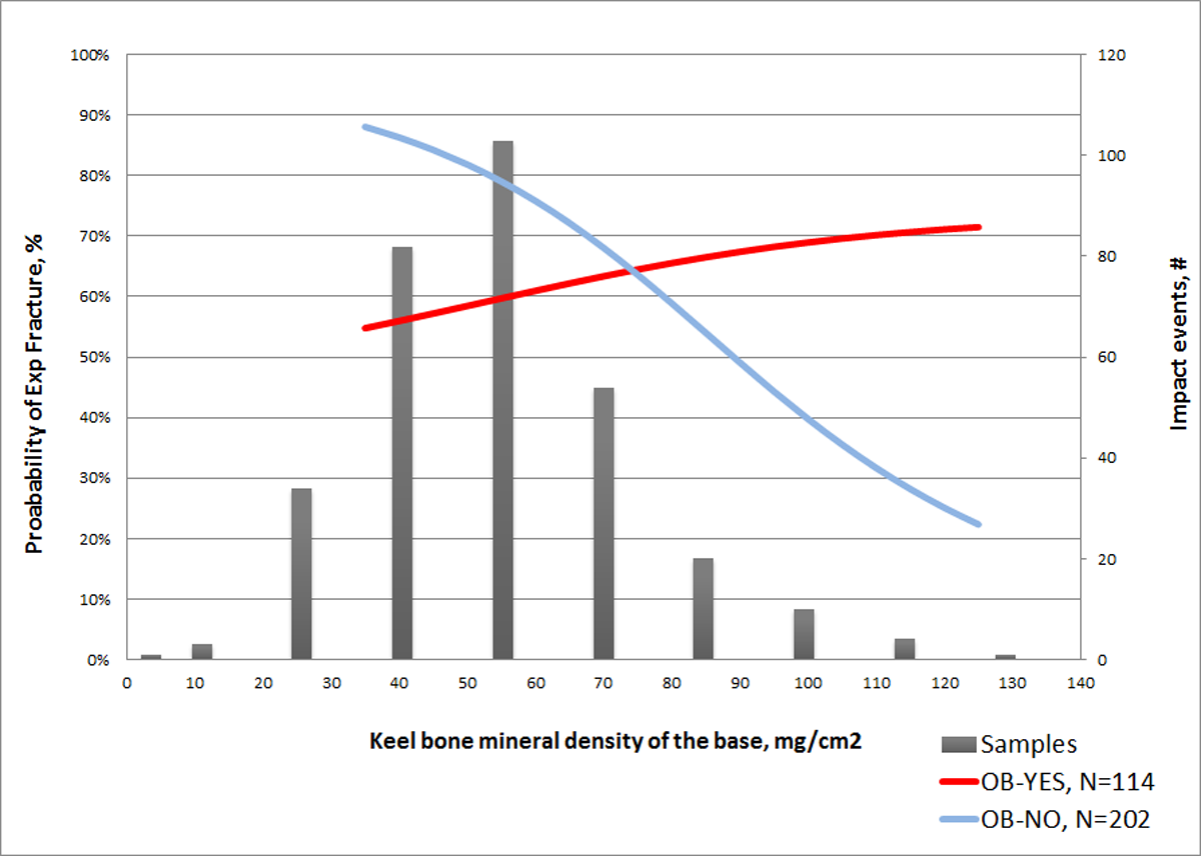
Modeled probability of an experimental fracture (%; Y-axis, left side) in relation to the mineral density of the keel’s base (mg/cm^2^; X-axis) and the presence of an old break. Superimposed on the graph is a histogram of the number of keels (#; Y-axis, right side) assessed.

**Fig 3:**
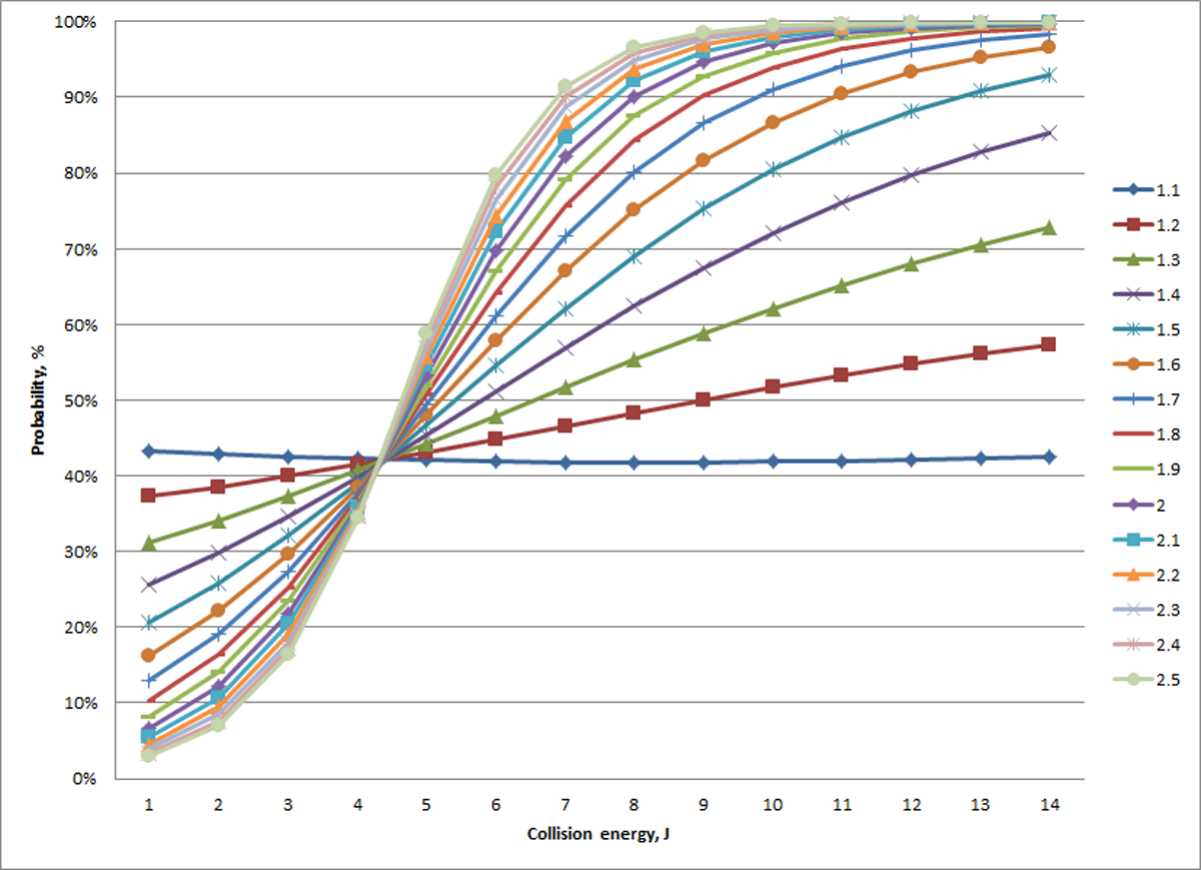
Modeled probability of an experimental fracture (%; Y-axis) in relation to impact collision energy (J; X-axis) and bird mass (Kg).

The best-fitting model defining the relationship between age and the likelihood of an experimental fracture occurring included a quadratic component for age and collision energy (Table 5). Visualization of the model across a range of collision energies and increasing age identified that probability of fracture initially increased with both collision energy and age (Fig 1). For age, the increased probability of fracture gradually flattened and reversed, i.e., older birds (calculated at greater than 49.5 weeks of age) had a decreased risk of fracture with increasing age. In the model assessing predictors other than age, experimental fractures were less likely to occur with increasing humerus strength (Table 5). The presence of an old break manifested an interaction with bone mineral density in the keel base where hens without an old break showed a relatively sharp fall in the likelihood of an experimental break as bone mineral density increased (Fig 2). Conversely, when an old break was present, the likelihood of experimental fracture increased with increasing bone mineral density, albeit a comparatively minor change in comparison to when an old break was absent. Collision energy and bird mass were also found to interact in terms of likelihood of an experimental fracture occurring (Table 5). At low bird masses, there was relatively no change across collision energies (Fig 3). With increasing bird mass, the probability of fracture exhibited a non-linear pattern with increasing collision energies, where those less than approximately 4.1 J manifested a disproportional response, i.e., increased bird masses had a decreased chance of experimental fracture at a given collision energy. In contrast, for collision energies greater than 4.1J, the probability of fracture increased with bird mass for a given collision energy.

**Table 5:** Model components for the binary response of whether an experimental fracture occurred or not in a model with predictors that included 1) collision energy and age only or 2) collision energy and selected non-age, biological factors. Odds ratios can not be provided for factors with model terms that involved a polynomial component or interaction.

	Binary fracture response				
	Age Focus		non-Age Focus		
Parameter	Estimate	SE	Estimate	SE	Odds Ratio
cons	-9.273	2.034	9.405	3.001	
Age	0.297	0.095			
Age^2^	-0.003	0.001			
Collision energy	0.737	0.169			
Collision energy^2^	-0.024	0.012			
Maxium load, Humerus			-0.016	0.004	0.9841
Old break presence			-3.673	1.809	
Keel bone mineral density, Base			-0.04	0.016	
Old break presence X Keel bone mineral density, Base			0.049	0.024	
Collision energy			-0.901	0.43	
Bird mass (Kg)			-3.503	1.647	
Collision energy X Bird mass (Kg)			0.812	0.262	

#### Severity response

**Fig 4:**
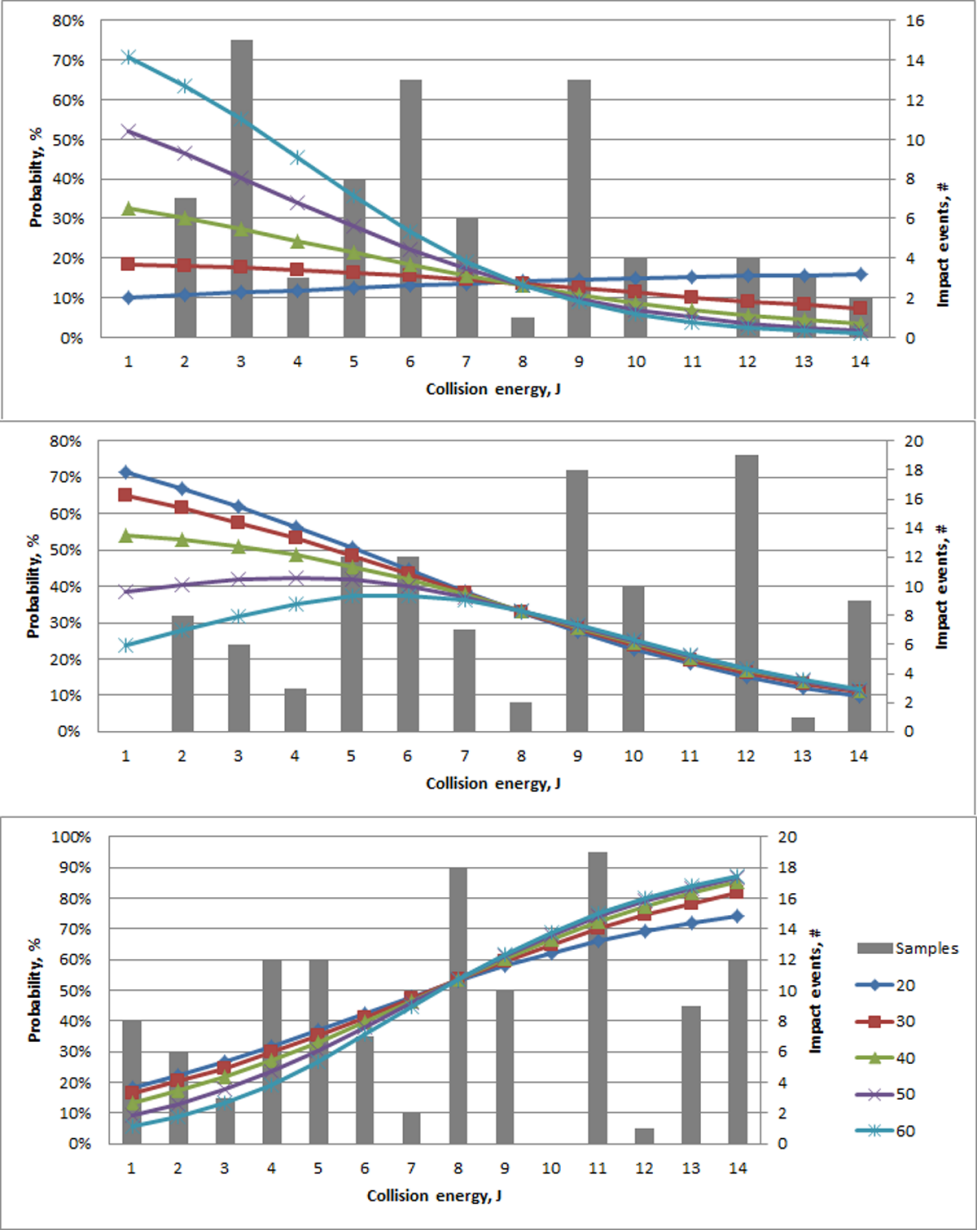
Modeled probability of experimental fracture (%; Y-axis,) type (Minor (top), Major (middle), and Severe (bottom)) in relation to collision energy (J; x-axis) and hen age (#; weeks represented by different line colors as show in the legend of the bottom panel). Superimposed on the graph is a histogram of the number of experimental impacts (#, Y-axis, rightside) across all ages that resulted within each respective severity grouping.

Age and collision energy were found to interact in terms of the likelihood of an experimental fracture occurring (Table 6). For collision energies less than 7.3 J, experimental fractures classified as Minor were more likely to occur in older animals, whereas the opposite was seen for Major experimental fractures (Fig 4). There also appeared to be differences in the variability across ages within a given collision energy. At collision energies greater than 7.3 J, the maximum difference between probabilities within a severity grade for a particular collision energy (14 J) was calculated as less than 14% (Minor experimental fractures, Fig 4 top panel). In comparison, the maximum difference for collision energies less than 7.3 J was 65% at 2J, also for Minor experimental fractures. Minor and Major experimental fractures were also far more variable across ages within a collision energy in comparison to Severe experimental fractures, particularly for collision energies less than 7.3 J.

**Table 6:** Model components for the response of experimental fracture severity with predictors that included 1) collision energy and age or 2) collision energy and all other non-age factors. Odds ratios are not provided for model terms that involved a polynomial component or interaction.

	Age		non-Age		
Parameter	Estimate	SE	Estimate	SE	Odds ratio
Constant (Major Fracture)	-2.982	1.641	-14.632	5.817	
Constant (Minor Fracture)	1.641	0.399	-0.375	4.003	
Age (Minor Fracture)	0.103	0.034			
Collision energy (Minor Fracture)	0.204	0.206			
Collision energy (Major Fracture)	-0.266	0.051			
Age * Collision energy (Minor Fracture)	-0.013	0.005			
Total energy, Humerus (Minor&Major Fracture)			-3.56	1.17	0.03
Modulus, Humerus (Minor&Major Fracture)			0.002	0.001	1.00
Breast mass (Minor Fracture)			0.082	0.027	1.09
Maxium load, Humerus (Minor Fracture)			0.013	0.005	1.01
Bird mass (Kg) (Minor Fracture)			1.778	3.804	
Bird mass (Kg) (Major Fracture)			2.312	2.299	
Collision energy (Minor&Major Fracture)			-0.05	0.107	
Collision energy^2^ (Minor&Major Fracture)			-0.039	0.008	
Maximum load, Humerus (Minor Fracture)			6.439	1.29	
Maximum load, Humerus (Major Fracture)			5.513	1.306	
Keel bone mineral density, Base (Major Fracture)			-0.073	0.017	
Bird mass (Kg) * Maximum load (Minor&Major Fracture)			-2.662	0.756	
Keel bone mineral density, Base * Collision energy (Major Fracture)			0.004	0.002	
Ca:P (Major Fracture)			2.738	0.719	
Ca:P (Minor Fracture)			0.635	0.278	
Ca:P * Bird mass (Kg) (Minor Fracture)			-1.605	0.442	
Ca:P * Bird mass (Kg) (Major Fracture)			-0.291	0.137	

**Fig 5:**
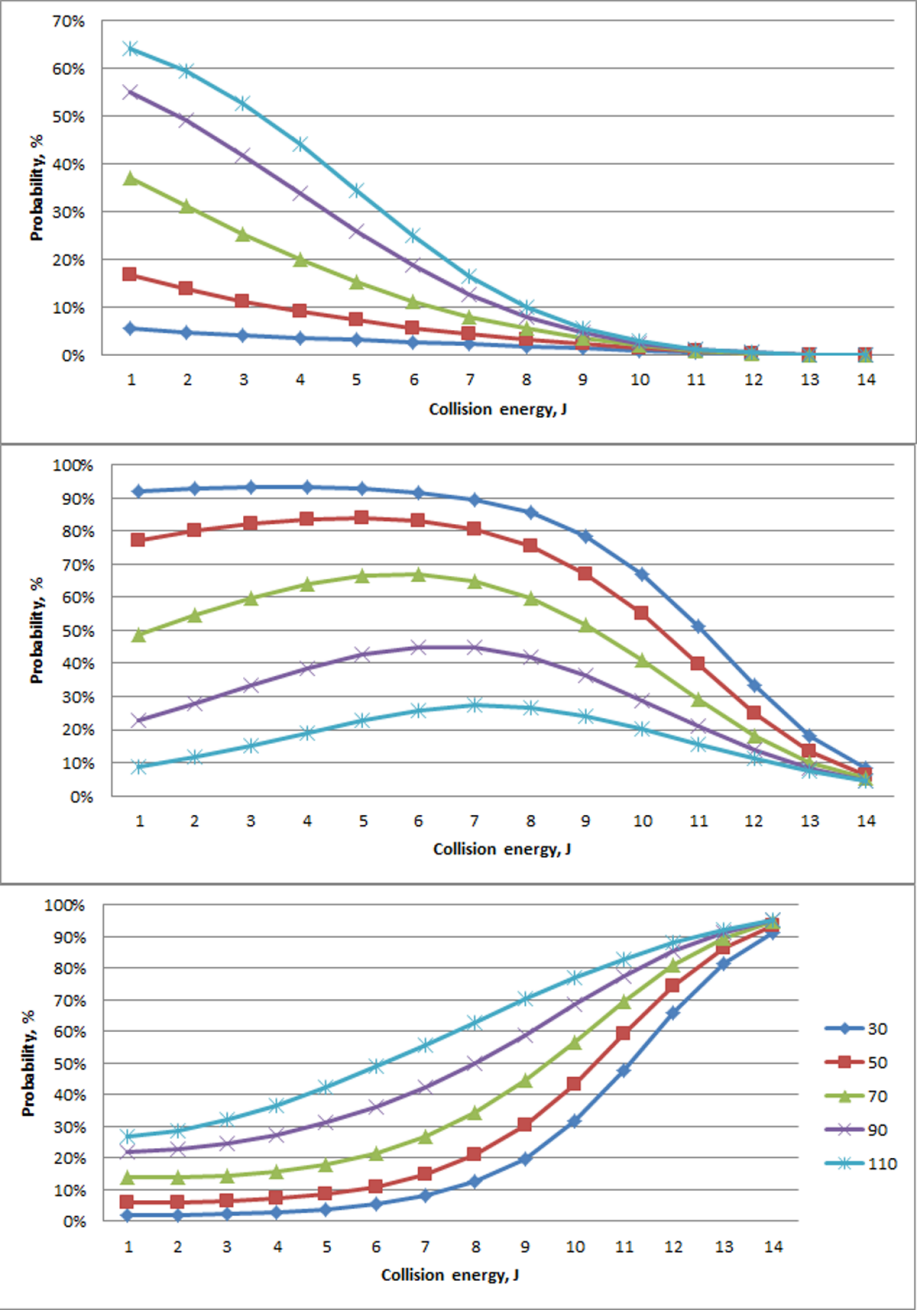
Modeled probability of experimental fracture (%; Y-axis,) type (Minor (top), Major (middle), and Severe (bottom)) in relation to collision energy (J; x-axis) and bone mineral density (mg/cm2) in the keel base.

In considering the selected non-age factors, multiple predictors were found to relate to the severity of experimental fracture (Table 6). As one example, bone mineral density interacted with collision energy where relatively lower collision energies (e.g., less than 8J) resulted in an increased probability of a Minor fracture as mineral density increased (Fig 5). In contrast, the opposite pattern in relation to bone mineral density was seen for Major experimental fractures.

**Fig 6:**
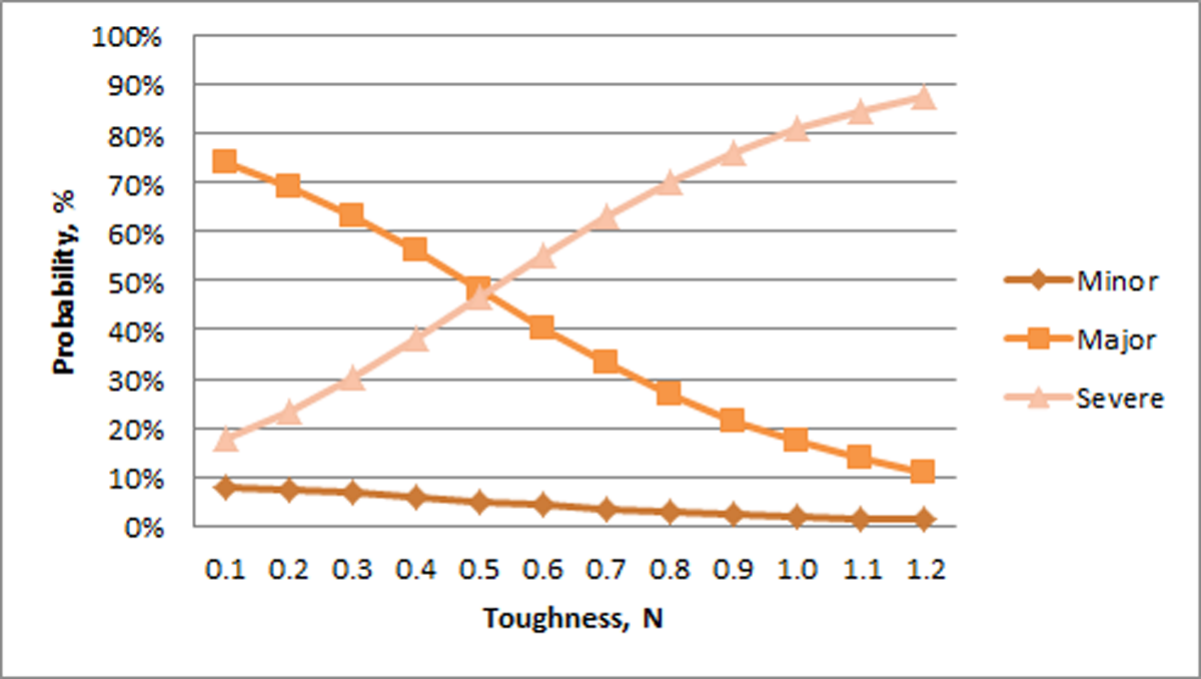
Probability of experimental fractures classified as Minor, Major, and Severe (%; Y-axis) in relation to toughness of the humerus (N; X-axis).

**Fig 7:**
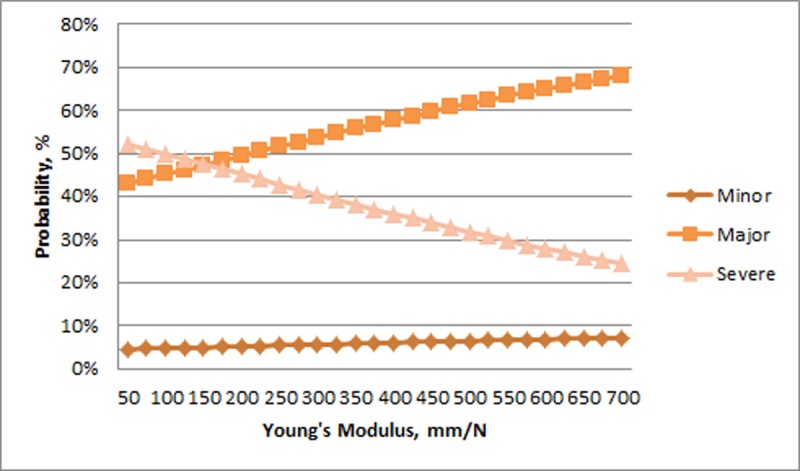
Probability of experimental fractures classified as Minor, Major, and Severe (%; Y-axis) in relation to Young’s Modulus of the humerus (mm/N; X-axis).

**Fig 8:**
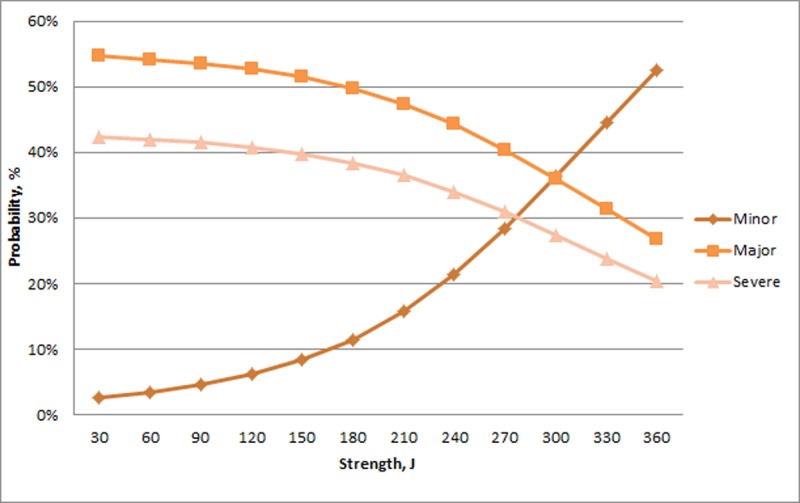
Probability of experimental fractures classified as Minor, Major, and Severe (%; Y-axis) in relation to humerus strength (J, X-axis).

The probability of Minor experimental keel fractures was relatively unchanged across the range of humerus toughness. In contrast, the probability of a Major experimental fracture decreased and the probability of a Severe experimental fracture increased with greater toughness (Fig 6). There was relatively little change across the range of stiffness for Minor experimental fractures (>8%) whereas there was a large increase in Major fractures and decrease in Severe fractures with increasing stiffness (Fig 7). For humerus strength, the probability of a Minor experimental fracture increased non-linearly from a relatively low value. The probability of Major and Severe fractures decreased with greater humerus strength (Fig 8).

**Fig 9:**
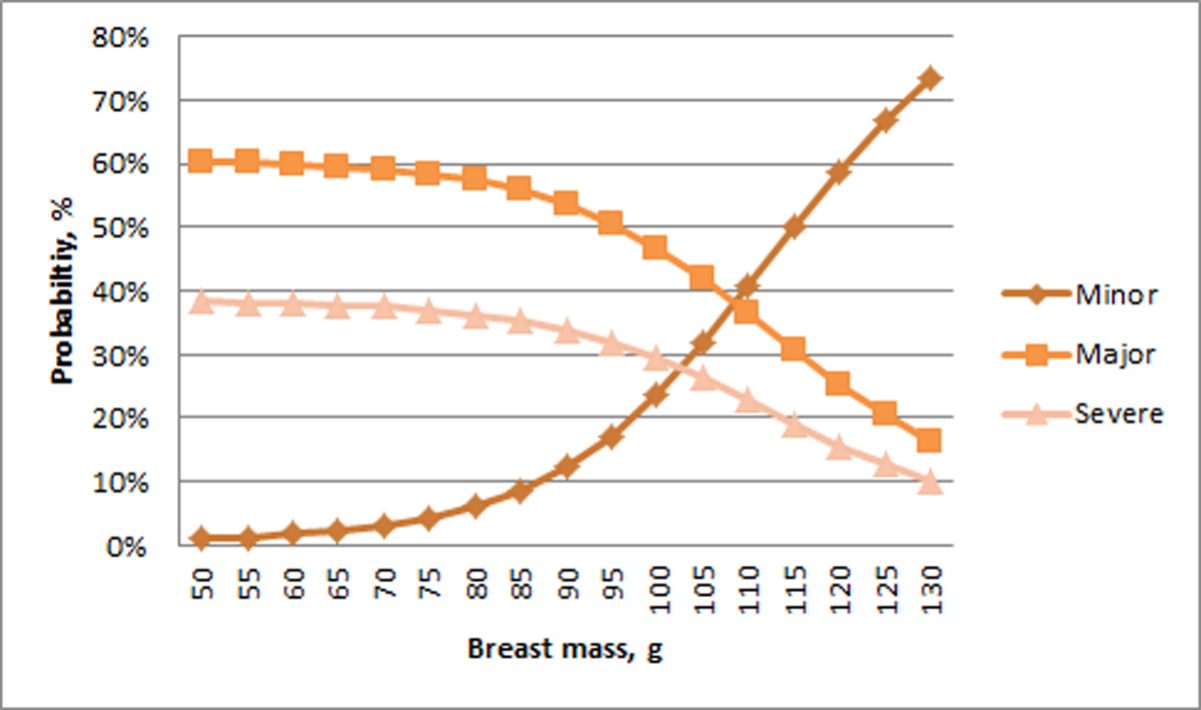
Probability of experimental fractures classified as Minor, Major, and Severe (%; Y-axis) in relation to breast mass (g, X-axis).

Regarding non-biomechanical properties, multiple predictors related to fracture severity (Table 6). The interaction of body mass and Ca:P related to fracture severity where Minor experimental fractures initially had a narrow range of probabilities that was proportional to bird mass (ranging from 5 to 40%) at the lowest Ca:P values (Table 6). As Ca:P values increased, the range of probability for Minor experimental fractures increased though maintained proportionality to bird mass. A similar pattern was seen in Severe experimental fractures, i.e. an initial relatively narrow range of probabilities at low Ca:P values that increased with Ca:P, though the probability of fractures occurring was inversely proportional to bird mass, i.e., heavier birds had a greater probability of Severe fractures at all Ca:P values. Low average breast muscle (less than 85 g) resulted in hens having predominantly Major (~60%) or Severe (~38%) experimental fractures (Table 6) (Fig 9). Above 85 g, the probability of Minor fractures began to increase rapidly with a concomitant fall in the values of Major and Severe experimental fractures.

### Omega-3 assessment

#### Impact testing

**Fig 10:**
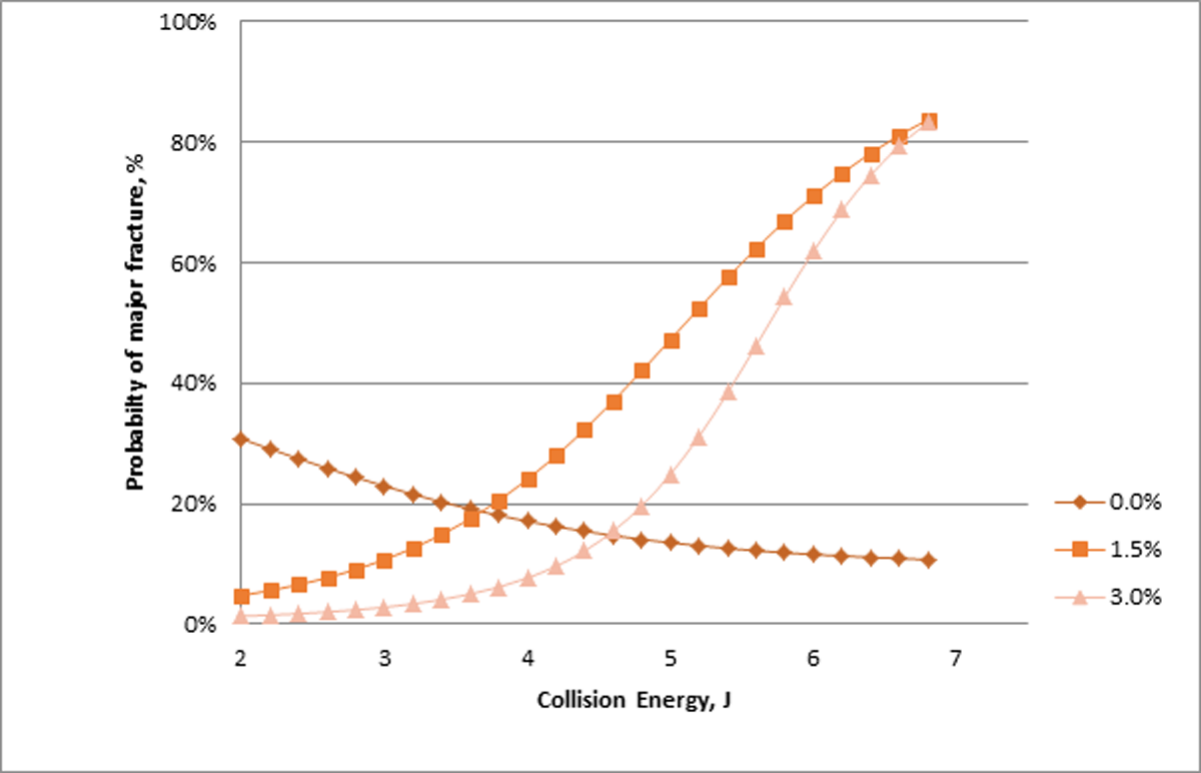
Probability of a Major experimental fracture occurring (%; Y-axis) across collision energy (J; X-axis) in relation to a minor fracture across treatment diet. The Figure represents both Major and Minor fractures, i.e. the proportion of Minor factures is the value of the line subtracted from one or the space above the line.

The testing protocol identified that the likelihood of experimental fracture occurring was affected by diet though depended on the quantity of collision energy. At low collision energies (at or below 3.2 J), the omega-3 diet appeared to afford a protective effect in terms of fracture incidence where the probability of a fracture occurring within this energy range was 40%, 34% and 21% for the 0.0%, 1.5% and 3.0% omega-3 diets, respectively. For collision energies above this threshold, the effect appeared to reverse where the probability of a fracture occurring was greater with an omega-3 diet, being 63%, 69%, and 79% for each diet respectively. Analysis of experimental fracture severity, i.e., comparing Major and Minor fractures, found a similar pattern to the occurrence of fractures where dietary omega-3 associated with a reduced probability of Major fracture at lower energies. The pattern reversed with increasing collision energy as the probability of a Major fracture increased (Fig 10). Above 3.2 J, the 3.0% diet maintained its protective effect compared with the 1.5% diet, i.e., Major fractures were more likely to occur in birds receiving the 1.5% diet. However, both omega-3 diets resulted in greater risk of experimental fractures compared with the control (0.0%) diet at higher collision energies. For the severity response, collision energy could be evaluated as a continuous variable allowing for a more precise determination of the response pattern over the energy range.

#### Bone health properties and bird mass

Young’s Modulus at the keel’s manubrial spine demonstrated a treatment effect independent of age where hens receiving the 3.0% omega-3 diet had reduced stiffness compared to the control. For comparison, we have also provided the respective values at both impact ages (Table 7). All other bone measures, including bone mineral density (g/m^2^; overall mean+/-SD: 7.44+/- 4.52), strength (N; overall mean+/-SD: 22.47+/- 5.32) and energy (J; overall mean+/-SD: 0.20+/- 0.06) at break failed to demonstrate a relationship with diet or age (p > 0.05). Bird mass did not show a diet effect (overall mean +/- SD: 1.76 +/- 0.15; p > 0.05).

**Table 7:** Stiffness of the base of the keel’s manubrial spine in relation to treatment diet. Age and treatment specific values are also provided, though there was no effect of age.

		Treatment diet					
		0.00%		1.50%		3.00%	
		Avg	StDev	Avg	StDev	Avg	StDev
Overall		427^a^	134	394^ab^	120	375^b^	122
Duration on diet, weeks							
	9	416	139	390	108	380	123
	18	443	130	400	138	370	123

Means that lack a common superscript are statistically different, p = 0.03.

#### Dietary intake

The ratio of feed consumption to egg production (gfeed/egg; overall mean+/-SD: 78.30+/- 11.75) and daily feed consumption per hen (g; overall mean+/-SD: 113.0+/-12.2) did not show a diet effect (p > 0.05).

#### Egg production

Egg production per hen (Hen day percentage) was maintained at a relatively high rate although no treatment differences were found (p> 0.05, overall mean: 95.4% ± 6.4). No treatment effect was found for egg weight (g; overall mean+/-SD: 62.3+/-3.8) or breaking strength (N; overall mean+/-SD: 49.7+/-7.5), and shell thickness (mm; overall mean+/-SD: 0.47+/- 0.12) or weight (g; overall mean+/-SD: 5.92+/-0.50) (p < 0.05).

## Discussion

### Experiment 1

A major obstacle in understanding the influencing factors of an event is the inability to visualize the underlying mechanism [19]. In the present context, our inability to visualize bone fractures in laying hens at the moment they occur and quantify potentially relevant factors (e.g. musculo-skeletal and biomechanical properties) is an impediment to understanding the problem of keel bone damage and providing solutions. The described impact testing protocol represents an effort to provide this information in the most biologically relevant manner possible. By creating controlled experimental conditions where impacts of variable collision energies can be delivered to the keel and the outcome assessed, we believe relevant factors can be isolated and used to direct future hypothesis-based testing and development of data-driven interventions. We have previously demonstrated the benefit of the impact testing method in comparing five distinct crossbred/purebred laying hen lines [13] and within a narrow range of ages, collision energies, and associated measures [12]. The current effort sought to build on this work and examine a wider range of collision energies, ages, and relevant bone properties in relation to experimental fracture.

#### Non-linear pattern of fracture likelihood in relation to age

The likelihood of experimental fractures followed a non-linear, age-dependent pattern that manifested a dramatic increase in the likelihood of fracture between 20 and 40 weeks of age. The increase within this age range has been reported in multiple reports examining on-farm commercial situations [3,5,7,20–22] and can likely be attributed to ongoing ossification of the keel as it transitions from a cartilaginous material to bone, a process reflected in biomechanical properties of the keel [7,23]. Unexpectedly, the likelihood of new fractures appeared to flatten in older hens, a pattern also reported in on-farm assessments of old breaks in several independent studies [3,7,21,24]. The reported pattern in older hens could previously be attributed to changes in behavior, e.g., improved ability to navigate within internal barn structures and avoid hazardous collisions, a factor supported by a correlation between internal barn complexity and old breaks in end-of-lay hens [1]. However, given the similarities between patterns observed on farm and the modeled data presented here where hens were impacted in a manner that excluded a direct behavioral role, we believe the decrease in likelihood of experimental fractures occurring is attributed to non-behavioral mechanisms, e.g. ongoing skeletal changes. Rath et al. [25] reported that, despite outward signs (e.g., weight, length, diameter) that the tibia of laying hens had ceased growing at 25 weeks of age, continuing changes in allometric, biochemical, and biomechanical properties that peaked at 35 weeks of age resulted in a relatively stronger and harder bone. In the current study, the increase in experimental fracture likelihood began leveling off and then reversed at approximately 49.5 weeks of age. The pattern observed in the current study roughly fits the observations reported by Rath et al. [25] assuming stronger and harder bone is resistant to fracture, though differences in exact timing should be investigated. It is likely that behavior maintains an indirect influence on the occurrence of fractures. For instance, birds that are more physically active would be expected to have increased bone loading which would lead to consequent skeletal changes such as greater strength in the short [23,26,27] and long [28] term. Nonetheless, our results strongly suggest that behavior cannot fully explain the observed fall in rate of new fractures for older hens.

Age was also associated with the severity of fractures where less severe fractures (i.e., Minor and Major) varied across age, particularly at a threshold of collision energies less than 7.3 J. Particularly for smaller collision energies, probabilities of Minor and Major experimental fractures opposed each other in relation to age. For example, in response to a 2J impact, the probability of a Minor experimental fracture increased with increasing age, whereas the opposite pattern was seen for a Major fracture. In other words, older birds were more likely to have a Minor fracture in response to a relatively small collision energy, a relationship that dissipated as collision energy increased and approached the approximate value of 7.3 J. Beyond 7.3 J, experimental fractures were increasingly scored Severe with increased collision energy. Our results suggest the mechanisms operating on fracture occurrence and severity are similar, e.g. mature bones of older birds are less likely to develop fractures and, when they do, are more likely to be minor in nature.

#### Relationship between measured properties of the keel and fracture susceptibility

Bone mineral density is often directly associated with biomechanical strength [29, 30] and linked with risk of fracture [31], hence a particular focus of the current work was the relationship between the mineral density and fracture susceptibility. Bone mineral density of the keel base related to both the occurrence and severity of fracture. In terms of severity, at relatively low collision energies, e.g less than 7 J, greater bone mineral density provided a protective effect with an increased probability of Minor experimental fractures occurring. With increasing collision energies, the protective effect dissipated with a rapid increase in the probability of a Severe experimental fracture occurring.

We also identified a relationship between likelihood of experimental fracture and the interaction of bone mineral density and the presence of an old break. In hens where an old break was absent, the probability of an experimental break occurring decreased with increasing mineral density, a relationship that fits our prediction where increased mineral provides greater strength and protection from fractures. In contrast, when old breaks were present, probability of experimental fracture increased with increasing bone mineral density, albeit with a considerably smaller rate of change (across density values) and from an initial higher probability. Taken together, the modeled response suggests that hens with relatively high bone mineral densities had greater fracture susceptibility if an old break was present. In other words, there was no protective effect of increased bone mineral density in hens when old breaks were present, and possibly a detriment. Direct biomechanical testing of the keel was not possible in Experiment 1, so we were unable to evaluate whether the increased bone mineral density provided structural benefits (e.g. strength) which could relate to altered fracture susceptibility. Nonetheless, it is interesting to consider the differing responses between hens with and without fractures. We would expect increased bone mineral in response to the healing process to stabilize the fractured components. However, increased mineral at isolated locations may not confer strength benefits for the entire keel despite similar densities in non-injured and injured keels. Future work should include measurements such as computed tomography or histology that allow for modeling of biomechanical properties (e.g., finite element analysis) across the length of the keel. It would also be helpful to quantify mineralization along the entirety of the keel’s transverse axis. Quantification using DEXA, as in the current study, provides only a gross quantity of mineral within the region of interest and does not differentiate locations within the keel, e.g., cortical vs. medullary bone. Given that cortical bone (vs. medullary) provides the structural strength to bone [32], increased mineral in medullary bone is unlikely to benefit fracture resistance and thus it would be important to differentiate between these two areas.

Structural characteristics of old breaks (e.g., fracture site, strength of the callus, size and orientation of the old break) are likely to also influence the outcome of an impact. For instance, the toughness of bone (amount of energy which can be absorbed before failure) is typically inverse to the elasticity [33]. If an old break in an advanced state of healing (i.e., relatively strong but inflexible) was caudal to the impact site, we could expect a new fracture to occur at the caudal boundary of the existing break. In support of this possibility, we often saw an experimental break in this fashion during the current work and have observed compound fractures (i.e., multiple juxtaposed, old break fracture lines in the transverse plane) during on-farm assessments (*unpublished observation*). Future work should assess relevant characteristics of old breaks and include means to ‘gauge’ the age of multiple fracture lines, i.e., juxtaposed fracture lines would be proportional in age along the cranial/caudal axis.

#### Relationships between experimental fracture susceptibility and non-keel parameters

Although properties of the keel were the principal focus of the impact testing protocol, our effort also allowed for tissues other than the keel to be assessed which can provide direction for intervention strategies. For instance, increasing humerus strength was associated with a decreased probability of experimental fracture and a greater probability of Minor fracture. The association between humerus biomechanical properties and experimental fracture occurrence/severity suggest a correlation between the structural properties of the humerus and the keel’s resistance to fracture. The relationship is not unexpected and has been reported previously [1,7,34], though the current work is the first that quantifies those relationships with the likelihood of a fracture occurring. By evaluating the measures side-by-side against the occurrence of whether a fracture actually occurs or not, we can better evaluate the relative benefits of focused breeding strategies.

Our effort also showed potential for properties in tissues other than bones to provide protective effects. Increasing breast mass correlated with an increased probability of Minor experimental fractures occurring. The mechanisms to explain this relationship will require further investigation, but larger muscles pulling on the keel could translate to greater loading and consequently increased strength. Alternatively, larger breast muscle could provide a stabilizing or protective effect at the moment of impact by absorbing impact energy. Lastly, breast muscle mass could be serving as a proxy for systemic bird health and reflect the overall condition of the bird, including bone health.

### Experiment 2

Following Experiment 1's general assessment of fracture susceptibility in relation to natural varying properties in animals across a range of ages, we sought to utilize the impact testing methodology to evaluate the benefit of a dietary intervention. Our results confirmed that fracture susceptibility varied with omega-3 content, though in a pattern where protective effects of treatment diets were limited to the relatively smaller collision energies. Previous efforts demonstrated reduced keel bone fracture with omega-3 enriched diets [6,7] and support the finding of reduced fracture, though do not explain the opposite effect at increased collision energies. Examination of bone quality and structure (*unpublished results)* from the hens assessed in Toscano et al. [7] indicated a reduced rate of bone turnover and a shift towards less mature bone with reduced presence of mature cross-links in hens receiving an omega-3 enriched diet. If a similar mechanism were operating within the current study, hens receiving the omega-3 diets would be expected to have weaker but more flexible keels and thus more prone to fracture at relatively higher collision energies. The notion of a more flexible keel is supported by the three point bending assessment of the modulus (in the keel) though no diet effect was seen for other biomechanical properties. Future work would benefit from a broader assessment of the keel properties including mineral content within the cortical bone as already discussed.

It is also interesting to consider the possibility that an intervention may be beneficial in some environments but detrimental in others. For instance, the omega-3 diet may be most beneficial for hens within cage systems where frequency and severity of fractures is known to be less than non-cage systems [1,3], likely as a result of smaller collision energies. Additional research is needed to investigate condition-specific solutions and under what circumstances benefits are best realized. The authors believe that the impact testing system would be an important tool in evaluating nutritional and other interventions in a standardized manner.

## General conclusions

Our study used an impact testing protocol to assess the relationship between likelihood of keel bone fracture and varying severity grades across a range of relevant bone and physiological measures. By eliminating behavioral confounds and allowing for visualization of the bone at the moment of impact our study effort provided a unique perspective on the factors which influence development of fractures and their severity. Most interestingly, in contrast to the predominant view that high egg production leads to increased fracture susceptibility, our results suggest that this relationship is likely valid only in the earliest stages of the lay period as fracture susceptibility fell from 49 weeks of age despite continued relatively high egg production. Additional information indicated that properties of other bones including humerus strength may serve as a proxy for keel health which could be a valuable tool for selective breeding efforts. A second study which sought to employ the impact testing procedure to evaluate a specific nutritional intervention provided positive results which suggested the omega-3 product has potential to reduce occurrence and severity of keel bone fractures, but likely only under relatively minor collision energies.

## Supporting information

S1 Database Microsoft Access database (KI.E1.mdb) containing all raw data for the Impact experiment. (MDB)

S2 Database Microsoft Access database (Alltech impact.use.accdb) containing all raw data for the Omega-3 experiment. (ACCDB)
